# Ruminal Protozoal Populations of Angus Steers Differing in Feed Efficiency

**DOI:** 10.3390/ani11061561

**Published:** 2021-05-27

**Authors:** Brooke A. Clemmons, Sung B. Shin, Timothy P. L. Smith, Mallory M. Embree, Brynn H. Voy, Liesel G. Schneider, Dallas R. Donohoe, Kyle J. McLean, Phillip R. Myer

**Affiliations:** 1 Department of Animal Science, University of Tennessee, Knoxville, TN 37996, USA; Brooke.Clemmons@tamuc.edu (B.A.C.); bhvoy@utk.edu (B.H.V.); lschneider@utk.edu (L.G.S.); kmclea10@utk.edu (K.J.M.); 2 U.S. Meat Animal Research Center, Clay Center, NE 68933, USA; Sung.Shin@ars.usda.gov (S.B.S.); Tim.Smith@ars.usda.gov (T.P.L.S.); 3 Native Microbials, San Diego, CA 92121, USA; mallory@nativemicrobials.com; 4 Department of Nutrition, University of Tennessee, Knoxville, TN 37996, USA; ddonohoe@utk.edu

**Keywords:** rumen, cattle, protozoa, microbiome

## Abstract

**Simple Summary:**

The rumen protozoa have been demonstrated to enhance methanogenesis and impact intraruminal recycling of microbial protein. However, they are also known to contribute to fiber degradation and the stabilization of ruminal pH changes. The apparent metabolic impact of ciliated protozoa in the rumen may contribute to the variation in feed efficiency. This study examined the relationship between the rumen protozoa and feed efficiency in beef steers. We monitored feed intake and body weight for a 70-day feed efficiency trial. Following the trial, rumen content was collected, protozoal DNA was extracted from the content, and the relationship between protozoa community diversity and species abundance with feed efficiency was examined. The ciliated protozoal community diversity differed between low- and high-feed efficient steers. Greater abundances of unidentified protozoa genera were detected in the low-feed efficient steers. These data suggest that unidentified protozoa and ciliated protozoal community diversity influence feed efficiency in beef steers.

**Abstract:**

Feed accounts for as much as 70% of beef production costs, and improvement of the efficiency with which animals convert feed to product has the potential to have substantial financial impact on the beef industry. The rumen microbiome plays a key role in determining feed efficiency; however, previous studies of rumen microbiota have not focused on protozoal communities despite the estimation that these organisms represent approximately 50% of rumen content biomass. Protozoal communities participate in the regulation of bacterial populations and nitrogen cycling—key aspects of microbiome dynamics. The present study focused on identifying potential associations of protozoal community profiles with feed efficiency. Weaned steers (*n* = 50) 7 months of age weighing approximately 260 kg were adapted to a growing ration and GrowSafe for 2 weeks prior to a 70-day feed efficiency trial. The GrowSafe system is a feeding system that monitors feed intake in real time. Body weights were collected on the first day and then every 7 days of the feed efficiency trial, and on the final day, approximately 50 mL of rumen content were collected via orogastric tubing and frozen at −80 °C. Body weight and feed intake were used to calculate residual feed intake (RFI) as a measure of feed efficiency, and steers were categorized as high (*n* = 14) or low (*n* = 10) RFI based on ±0.5 standard deviations about the mean RFI. Microbial DNA was extracted, and the eukaryotic component profiled by amplification and sequencing of 18S genes using degenerate primers that can amplify this locus across a range of protists. The taxonomy of protozoal sequences was assigned using QIIME 1.9 and analyzed using QIIME and SAS 9.4 with significance determined at α ≤ 0.05. Greater abundances of unassigned taxa were associated with high-RFI steers (*p* = 0.03), indicating a need for further study to identify component protozoal species. Differences were observed between low- and high-RFI steers in protozoal community phylogenetic diversity, including weighted beta-diversity (*p* = 0.04), Faith’s phylogenetic diversity (*p* = 0.03), and observed Operational taxonomic unit (OTU) (*p* = 0.03). The unassigned taxa and differences in phylogenetic diversity of protozoal communities may contribute to divergences observed in feed efficiency phenotypes in beef steers.

## 1. Introduction

The rumen microbiome is key to effective feed degradation and utilization by the host animal, ultimately affecting its nutritional status. The rumen microbiota of cattle are estimated to produce approximately 70% of the total energy substrates absorbed and used by the animal. Much of the research conducted to understand the relationship between the ruminant host and the rumen microbiome has focused on bacterial communities. Bacteria are arguably the most diverse microbial kingdom in the rumen and are the most abundant microbes. Ruminal bacteria provide many critical functions in support of ruminant nutrition and health, such as proteolytic and fibrolytic activities and vitamin synthesis. Due to these important roles, rumen microbiome research has historically focused on bacterial communities heavily [1,2,3]. However, protozoa represent an approximately equal volume of rumen biomass even though they have lower total cell number than bacteria [4]. Importantly, predation of ruminal bacteria by protozoa and the resulting protein degradation is a major component of nitrogen use efficiency in ruminants [5]. Approximately 65% of the dietary N is converted to microbial protein, thus providing high-quality protein to the host [6]. This breakdown of microbial protein ultimately results in the production of oligopeptides and free amino acids, which are rapidly fermented to short-chain fatty acids and ammonia, resulting in the intraruminal nitrogen cycling commonly known to cause low nitrogen use efficiency in ruminants [4,5,6]. However, rumen protozoa can exhibit positive effects on the rumen ecosystem. Specifically, rumen ciliate protozoa can engulf and hoard starch, store the granules as glycogen, and hydrolyze and ferment them to volatile fatty acids (VFAs) [4,6]. This starch hoarding by rumen protozoa has been hypothesized to help stabilize the rumen ecosystem and improve its resilience to pH fluctuations.

Much of the role of protozoa in ruminal fermentation, host metabolism, and feed efficiency has yet to be elucidated. Numerous sources of variation in feed efficiency have been theorized and validated, such as animal genetics, nutrition, physiology, host and microbial metabolism, and animal behavior [7]. However, considerable variation has also been attributed to differences in nutrient utilization by bacteria and ruminal microbes among cattle [3,8,9,10,11]. Rumen protozoa have been demonstrated to impact nitrogen utilization, the metabolic and functional potential of the rumen, and the stability of the rumen microbial ecosystem. Given this functional importance of ciliate protozoa within the rumen environment and the symbiotic and predatory relationships among all domains of rumen microbes [6], it is critical to not only categorize the diversity and phylogeny of rumen protozoa, but also determine their association with nutrition and feed efficiency in beef cattle. The objective of this study was to characterize the protozoal community in the rumen from steers differing in feed efficiency using deep 18S ribosomal RNA gene (18S rDNA)-based community profiling. We hypothesized that variation in the protozoal populations within the rumen could contribute to variation in feed efficiency.

## 2. Materials and Methods

This study was approved and carried out in accordance with the recommendations of the Institutional Animal Care and Use Committee at the University of Tennessee, Knoxville, approval code 2466-0616.

### 2.1. Animals, Experimental Design, and Rumen Content Sampling

The study was conducted at the University of Tennessee Institute of Agriculture Plateau Research and Education Center in Crossville, TN. Fifty purebred Angus steers, weighing 264 ± 2.7 kg and 7 months of age at the start of the trial, were used for the study. Two weeks post-weaning and 10 days prior to measuring feed intake, the steers were acclimated to the GrowSafe individual feed intake monitoring system (GrowSafe Systems Ltd., Airdrie, AB, Canada). The steers were fed a step-up receiving diet for 14 days before beginning the trial with a growing ration (11.57% crude protein and 76.93% total digestible nutrients on a dry matter (DM) basis) with 28 mg monensin/kg DM [11]. Following the acclimation period, feed intake was precisely measured using the GrowSafe system, and body weight [12] was measured at 7 day intervals for a 70 day feed efficiency trial. At the end of the feeding trial, the steers were ranked by residual feed intake (RFI; the difference between actual dry matter intake (DMI) and expected DMI) as a measure of feed efficiency, based on performance and feed intake measured from day 0 to day 70 [13]. High-RFI animals are less efficient, while low-RFI animals are more efficient. The average value of RFI and standard deviation (SD) were calculated for each individual animal. High-RFI (*n* = 14) animals were identified as an RFI ≥ 0.5 SD above the mean, and low-RFI (*n* = 10) steers were determined by an RFI ≤ 0.5 SD below the mean. At day 70, approximately 50 mL of rumen content was sampled via orogastric tubing, which has been shown to be a practical sampling method to capture a comprehensive rumen microbial community, especially on higher grain diets [14].

### 2.2. DNA Extraction, Amplification, and Sequencing

Microbial DNA from the low- and high-RFI steers was extracted using the rumen fiber and fluid content samples via the repeated bead beating plus column digesta extraction protocol [15] and modified similar to that of Myer and colleagues [10]. Metagenomic DNA concentration was determined using the DeNovix DS-11 Series Spectrophotometer/Fluorometer (DeNovix, Inc., Wilmington, DE, USA). The DNA samples were stored at −20 °C until amplification and library preparation.

Amplicon library preparation was performed by PCR amplification encompassing variable regions V3 and V4 and rumen ciliate signature regions 1 and 2 of the 18S rRNA gene. Specifically, the amplicon libraries were constructed using the modified primer set P-SSU-316F (5′ adaptor/index/GCTTTCGWTGGTAGTGTATT) and GIC758R (5′ adaptor/index/CAACTGTCTCTATKAAYCG) [16], including adapter sequences and custom indices, as well as AccuPrime Taq high-fidelity DNA Polymerase (Life Technologies, Carlsbad, CA, USA). The following conditions were used for PCR amplification: initial denaturation step at 95 °C for 3 min, followed by 35 cycles of denaturation at 95 °C for 30 s, annealing at 55 °C for 30 s and extension at 72 °C for 2 min, and a final extension step at 72 °C for 7 min. Amplicons were purified using AMPure XP bead purification (Agencourt AMPure, Beckman Coulter, Danvers, MA, USA), and all libraries were quantified using the PicoGreen dsDNA quantitation kit (Invitrogen Corp., Carlsbad, CA, USA) and by real-time PCR on the LightCycler 480 System (Roche Diagnostics, Mannheim, Germany). The amplicon libraries were sequenced using the v3 2 × 300 kit and the Illumina MiSeq sequencing platform (Illumina, Inc., San Diego, CA, USA).

### 2.3. Sequence Analysis

Protozoal 18S amplicon sequence reads were processed using the Quantitative Insights Into Microbial Ecology (QIIME) bioinformatics pipeline, version 1.9.1 [17]. Adapters/index sequences were trimmed, and sequences were removed if they had an average quality score < Q30. Chimeric sequences were identified and filtered using usearch61 [18]. Operational taxonomic unit (OTU) picking was completed utilizing the cleaned sequences and was clustered with a pairwise identity threshold of 97%, and further assigned to taxonomy using the Silva reference database, 128 release [19].

### 2.4. Volatile Fatty Acids

The rumen samples were prepared for VFA analysis through centrifugation of the strained samples at 10,000× *g* for 10 min at 4 °C. A mixture was then prepared consisting of 5 mL of rumen fluid supernatant and 1 mL of meta-phosphoric acid-2ethyl butyric acid solution. This mixture was placed in an ice bath for ≥30 min and was then centrifuged for 10 min at 10,000× *g* and 4 °C. Using the previously described method [20], the samples were analyzed using gas chromatography (Agilent 7890B, Agilent Technologies, Inc., Santa Clara, CA, USA). The gas chromatograph was equipped with an FID detector, Nukol fused silica capillary column (Supelco, Sigma-Aldrich Co., LLC, Bellefonte, PA, USA), and helium as the carrier gas.

### 2.5. Statistical Analysis

Protozoal OTU were analyzed in SAS 9.4 (SAS Institute, Cary, NC, USA) and QIIME version 1.9.1 [17]. Alpha-diversity metrics, including Good’s coverage, chao1, Faith’s phylogenetic diversity, observed OTU, Shannon’s diversity index, and Simpson’s evenness E, as well as genus-level taxa and VFA, were evaluated for normal distribution in SAS 9.4 using the PROC UNIVARIATE procedure. Normality was determined based on a Shapiro–Wilk statistic ≥0.85 and visualization of histograms. Variables that followed a normal distribution were analyzed using a one-way analysis of variance (ANOVA). Those variables not following a normal distribution were first log-transformed or ranked to achieve normality, and then analyzed using a one-way ANOVA. Multiple test correction was applied using false discovery rate (FDR) corrected *p*-values. Beta-diversity was assessed using analysis of similarity (ANOSIM) and permutational multivariate analysis of variance (PERMANOVA). Relationships between protozoal taxa and VFA were analyzed using Spearman correlation with the PROC CORR procedure in SAS 9.4. Statistical significance was determined using α = 0.05.

## 3. Results

### 3.1. Sequence Data, Alpha-Diversity, and Beta-Diversity of Protozoal Communities

A total of 13,793,949 reads remained for subsequent analyses after quality filtering and chimera detection and filtering. From the total cleaned reads, after OTU clustering where OTU were defined as a read sharing ≥97% nucleotide sequence identity, a total of 7237 OTU were identified across all samples.

Protozoal alpha-diversity was examined using microbial community diversity parameters of observed richness (observed OTU), estimated richness (chao1), richness and evenness (Shannon), coverage (Good’s Coverage), phylogenetic diversity (Faith’s phylogenetic diversity), and evenness (Simpson’s evenness E). Two alpha-diversity metrics, observed OTU (*p* = 0.03) and Faith’s phylogenetic diversity (*p* = 0.03), differed by RFI (Table 1). None of the other alpha-diversity metrics were different between high- and low-RFI steers. Each RFI group was adequately covered at 99% coverage (Good’s coverage), and coverage did not differ between groups.

**Figure 1:**
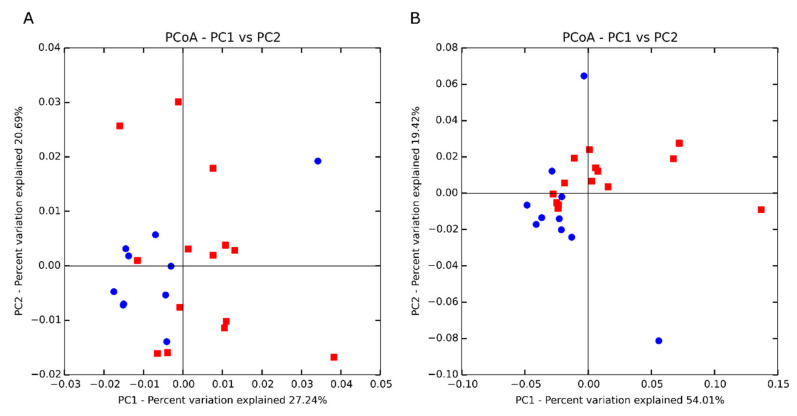
Principal coordinates analysis based on unweighted (**A**) and weighted (**B**) UniFrac distances of different levels of residual feed intake based on 9999 permutations. Low RFI = blue circle, High RFI = red square.

Beta-diversity was visualized using principal coordinates analyses (PCoA) based on unweighted (presence/absence of different taxa; Figure 1A) and weighted (abundances of different taxa; Figure 1B) UniFrac distances. Marginal separation into clusters was observed in the weighted PCoA, as supported by weighted beta-diversity analyses ANOSIM and PERMANOVA. Beta-diversity was analyzed using ANOSIM and PERMANOVA, and divergence was observed by RFI in weighted analyses with significance between high- and low-RFI steers using weighted PERMANOVA (*p* = 0.04). Although not significant, trending differences between high- and low-RFI steers were also identified using weighted ANOSIM (*p* = 0.07; Table 2).

### 3.2. Taxonomic Composition

The 13,793,949 cleaned reads were classified into five genera, including *Diplodinium, Entodinium, Isotricha, Ophryoscolex,* and *Trichostomatia.* Approximately 4% of the reads could not be assigned to a known genus. Taxa were deemed nondetectable at abundances ≤0.0001%. No classified genus-level protozoal taxa abundances were different between high- and low-RFI steers (Table 3). The sole classification that differed by RFI following FDR correction was unassigned taxa (*q* = 0.03), though there were large numerical differences in other genera by RFI (Table 3).

### 3.3. Ruminal VFA Proportions

The proportions of ruminal acetate, propionate, isobutyrate, butyrate, and valerate (Table 4) did not differ between high- and low-RFI animals. Although no differences were identified in VFA proportions, there were several significant correlations observed between protozoal taxa and VFA (Table 5).

## 4. Discussion

Beef consumption is increasing globally as beef provides an excellent source of high-quality protein [21]. The United States is one of the largest exporters of beef, exporting more than 1 million tons of beef annually [21,22]. In the United States beef industry, feed costs account for more than 70% of the total input costs of production [22,23,24]; thus, identifying methods for improving feed efficiency would help to decrease production costs and provide greater quantities of high-quality protein available for global consumption. The rumen microbiome contributes to the variation in feed efficiency phenotypes [3]; however, much of the research examining feed-efficiency-associated rumen microbial communities is related to bacteria and archaea, likely due to their functional and genetic diversity, high abundance, and/or contribution to greenhouse gas production. Much less is understood about the specific relationship between rumen ciliate protozoa and feed efficiency in cattle despite research demonstrating their roles in nitrogen balance, microbial protein synthesis, and average daily gain [6,25]. Therefore, this study examined the relationship between rumen protozoal communities and feed efficiency in beef cattle.

Several measures of alpha- and beta-diversity differed by RFI in this study. The metrics that differed were those related to phylogenetic diversity, including Faith’s phylogenetic diversity, number of observed OTU, and weighted UniFrac distance metric, with greater phylogenetic diversity observed in low-RFI steers. These results are antithetical to those regularly observed in bacterial communities, in which bacterial diversity does not typically differ by RFI [2,3]. However, greater ecosystem diversity on a macro scale tends to result in greater adaptability and resilience of an ecosystem [26]; in particular, functional diversity leads to a more productive and efficient ecosystem [27]. In the rumen, greater phylogenetic diversity of protozoa may be reflective of greater functional diversity given the variation in substrate utilization and metabolism [6,28]. Increased protozoal diversity has the potential to increase substrate availability to other microbiota and the host, and may help to mitigate the negative effects of nitrogen turnover by protozoal communities [6]. Defaunation studies have demonstrated that when protozoa are removed from the ruminal environment, the structure of the bacterial community is impacted, resulting in reduced bacterial diversity [5,29]. This lack of diversity as a result of defaunation has also been corroborated by meta-analyses indicating reductions of important fibrolytic microorganisms, including anaerobic fungi and the abundant bacteria *Ruminococcus albus* and *Ruminococcus flavefaciens* [6]. Increases in protozoal diversity may therefore be indicative of increases in the abundance and diversity of fibrolytic microbes and improvements in fiber digestion, resulting in improved feed efficiency.

The only classification that differed by RFI in this study were the unassigned taxa. This difference is a trend also observed in bacterial communities, where low- and high-RFI cattle have differed with regard to unassigned bacteria relative abundances [2]. There was a greater abundance of unassigned protozoal taxa in high-RFI steers compared with low-RFI steers. These unassigned taxa may provide great insight into divergences in feed efficiency phenotypes in ruminants, particularly given that there is increasing evidence that rare or low-abundance taxa may drive important phenomena in other ecosystems [30,31,32]. For instance, Aanderud and others examined rare taxa in various ecosystems before and after a soil rewetting event [33]. The authors established that following the rewetting events, formerly rare taxa often became dominant, sometimes accounting for up to 60% of relative abundance of bacteria [33]. Additionally, those bacterial community changes were associated with drastic alterations in gas production, with up to 20-fold increases in CO_2_ production and 150% reduction in methane production [33]. The same impacts of rare microbial taxa have been observed in numerous organisms and ecosystems [32,34,35], highlighting the importance of understanding the effects that rare microbial taxa may have on their hosts via changes in microbial diversity and community structure.

There were three times as many *Diplodinium* in the high-RFI steers compared with low-RFI steers, although the difference was not significant after correction for FDR. *Diplodinium* possesses cellulolytic capabilities that are not represented by all ruminal ciliates and appears to perform better in the rumen of animals consuming greater proportions of forage in the diet. Eun et al. examined the effect of increasing concentrate in the diet on protozoal populations in sheep, with concentrate ranging from 10% to 70% of the diet [36]. Abundance of *Diplodinium* increased in sheep fed diets consisting of up to 40% concentrate; however, at concentration inclusion rates of 50% or greater, abundances of *Diplodinium* decreased [36]. The steers in the present study were fed a primarily corn-silage-based growing ration, and the greater proportion of readily digestible feedstuffs may not have been an optimal diet for *Diplodinium* communities, contributing to their variability among animals and lack of differences contrasted with other research. The relative dominance of *Diplodinium* in high-RFI steers may also have contributed to the noted differences in phylogenetic diversity in high-RFI steers, potentially resulting in a less efficient ruminal microbiome.

Protozoal engulfment of starch granules and subsequent fermentation to VFA has been theorized to help stabilize the rumen ecosystem and its resilience to pH fluctuations [4,6]. These associations between significant populations of protozoa and VFA are reflected in the correlations between highly abundant protozoa genera and VFA in the current study. Specifically, members of both *Diplodinium* and *Entodinium* were correlated with butyrate. The genus *Entodinium* is the most abundant genus of protozoa, accounting for >95% of the total rumen, or averaging approximately 76% in this study. The associations between butyrate and *Entodinium* and its impact in this study may be based on the noted high abundance of this genus, as the absence of protozoa via defaunation has been shown to decrease ammonia and butyrate production in vitro and in vivo [37,38]. The shift of VFA profiles towards greater propionate and less acetate and butyrate in response to defaunation has also been commonly demonstrated to reduce total VFA concentrations and decrease dietary fiber digestibility, signifying that rumen protozoa contribute to fiber digestion [38]. Further, this decrease in digestibility is likely directly related to fiber degradation and not a pH-dependent effect, as meta-analyses have demonstrated limited impacts of defaunation on ruminal pH [6]. Although these decreases in rumen fiber digestion as a result of defaunation may be small and moderately compensated by the increase of degradation in the hindgut [39], the noted differences in ruminal nutrient availability may contribute to divergences in feed efficiency phenotypes in ruminants.

The correlation of many protozoa with butyrate or isobutyrate is also significant due to the known association of ruminal butyrate concentrations with highly efficient animals [40]. Numerous species of microbes in the rumen dynamically degrade starch and utilize its intermediates. These varieties of microbes typically have fast growth rates, quickly fermenting starch and/or soluble sugars contributing to accumulations of VFA. In extreme examples of rapid starch utilization, large increases in butyrate concentrations were observed when animals were intraruminally supplemented with starch to induce acidosis and when lactic acid accumulation was prevented with ionophores such as monensin [41,42]. The use of monensin in the diet in the current study and increased proportions of ruminal butyrate is consistent with the aforementioned studies. Butyrate is known to contribute greatly towards cattle energy requirements compared with acetate [43]. Further, microbial activity resulting in butyrate production instead of acetate is known to divert H_2_ from methanogenesis, improving feed efficiency. This is confounded, however, by the known (endo)symbiotic and catalytic roles protozoa play with methanogens and ruminal methanogenesis [44]. Defaunation studies have demonstrated that the removal of protozoa from the rumen results in decreased methane production [6,37], explaining approximately 47% of the variability in methane emissions [45]. The role of protozoa in this context of efficiency and nutrient utilization within the ruminal microbial ecosystem remains unclear [6].

## 5. Conclusions

The present study is one of very few that examine the relationship between feed efficiency and rumen protozoal communities. Protozoal communities are important members of the rumen microbiome, accounting for approximately 50% of the microbial biomass. The present research provides preliminary insight into the relationship between the rumen protozoa and feed efficiency in beef cattle. Additional research should focus on further understanding the relationship between the protozoal communities and feed efficiency in ruminants. Specifically, determining how feed efficiency impacts ruminal protozoal nitrogen outflow could aid in further understanding protozoal impacts on host ruminant metabolism and define how protozoa contribute to the variation in feed efficiency.

## Figures and Tables

**Table 1:** Alpha-diversity metrics between high- and low-residual feed intake (RFI) steers.

Metric	High RFI ^1^	Low RFI ^1^	*p*-Value ^2^	FDR ^2,3^
Good’s coverage	0.99 (0.00)	0.99 (0.00)	0.03	0.07
Observed OTU (Operational taxonomic unit)	297.93 (1.79)	306.60 (2.71)	0.01	0.03
Faith’s phylogenetic diversity	142.11 (0.44)	144.42 (0.74)	<0.001	0.03
Chao1	310.11 (2.64)	315.01 (1.08)	0.15	0.22
Shannon’s diversity index	1.86 (0.12)	1.77 (0.21)	0.69	0.69
Simpson’s evenness E	0.0074 (0.00)	0.0079 (0.00)	0.67	0.69

^1^ Mean (SEM) Standard error of the mean. ^2^ Significance determined at *p* ≤ 0.05. ^3^ False discovery rate corrected *p*-value.

**Table 2:** Beta-diversity analyses between high- and low-residual feed intake (RFI) steers.

Metric ^1^	Test Statistic	*p*-Value
PERMANOVA ^2^-weighted	2.76 ^4^	0.04
PERMANOVA ^2^-unweighted	0.80 ^4^	0.58
ANOSIM ^3^-weighted	0.11 ^5^	0.07
ANOSIM ^3^-unweighted	−0.06 ^5^	0.85

^1^ Metric analyses based on weighted or unweighted UniFrac distances with 9999 permutations. ^2^ Permutational multivariate analysis of variance. ^3^ Analysis of similarity. ^4^ Test statistic is pseudo-F. ^5^ Test statistic is R.

**Table 3:** Genus-level taxon differences between high- and low-residual feed intake (RFI) steers.

Genus	High RFI ^1^	Low RFI ^1^	*p*-Value ^2^	FDR ^2,3^
*Diplodinium*	0.12 (0.06)	0.04 (0.03)	0.05 ^4^	0.15 ^4^
*Entodinium*	0.67 (0.09)	0.81 (0.07)	0.13 ^4^	0.19 ^4^
*Isotricha*	0.06 (0.06)	0.05 (0.05)	0.28 ^4^	0.33 ^4^
*Ophryoscolex*	0.08 (0.06)	0.02 (0.02)	0.13 ^4^	0.19 ^4^
*Trichostomatia*	0.02 (0.01)	0.05 (0.03)	0.74 ^5^	0.74 ^5^
Unassigned	0.05 (0.00)	0.03 (0.00)	<0.001 ^4^	0.03 ^4^

^1^ Mean (SEM) Standard error of the mean. ^2^ Significance determined at *p* ≤ 0.05. ^3^ False discovery rate corrected *p*-value. ^4^ Based on ranked data. ^5^ Based on log-transformed data.

**Table 4:** Relative proportions of volatile fatty acids by high- and low-residual feed intake (RFI) steers.

VFA ^1^	High RFI ^2^	Low RFI ^2^	*p*-Value ^3^
Total ^4^	26.35 (2.39)	31.49 (2.38)	0.15
Acetate	63.88 (1.37)	63.09 (1.33)	0.69
Propionate	8.62 (0.89)	7.47 (0.98)	0.39
Isobutyrate	0.37 (0.04)	0.42 (0.04)	0.33
Butyrate	25.43 (1.22)	26.01 (1.69)	0.78
Valerate	1.69 (0.42)	3.00 (0.88)	0.16

^1^ Volatile fatty acids (VFAs) expressed as mM × 100 mM^−1^, except where otherwise noted. ^2^ Mean (SEM) Standard error of the mean. ^3^ Significance determined at *p* ≤ 0.05. ^4^ Concentration in mM.

**Table 5:** Significant correlations between genera abundances of rumen protozoa and volatile fatty acid proportions.

Taxon	Metabolite	R^2^	*p*-Value ^1,2^
*Trichostomatia*	Propionate	−0.41	0.05
*Ophryoscolex*	Isobutyrate	−0.44	0.03
*Ophryoscolex*	Butyrate	0.43	0.03
*Diplodinium*	Butyrate	0.55	<0.01
*Entodinium*	Butyrate	−0.55	<0.01

^1^ Significance determined at *p* ≤ 0.05. ^2^ Based on Spearman correlation.

## Data Availability

The data presented in this study are available on request from the corresponding author. The data are not publicly available due to restrictions.
